# Supplementary material for: Identification of photoperiod-induced specific miRNAs in the adrenal glands of Sunite sheep (Ovis aries)
Source: Front Vet Sci. 2022 Jul 22;9:888207. doi: 10.3389/fvets.2022.888207 (PMC9354845; doi:10.3389/fvets.2022.888207)
Supplement: Supplementary file 1 [file Data_Sheet_1.ZIP › Supplementary file/miRDeep2 file.zip!/miRDeep2 file/Novel_Structure/SPLP42A3/Novel_643.pdf]

Provisional ID	:	NC_019459.2_4074
Score total	:	2.6
Score for star read(s)	:	-1.3
Score for read counts	:	0
Score for mfe	:	2.3
Score for randfold	:	1.6
Score for cons. seed	:	
Total read count	:	15
Mature read count	:	15
Loop read count	:	0
Star read count	:	0

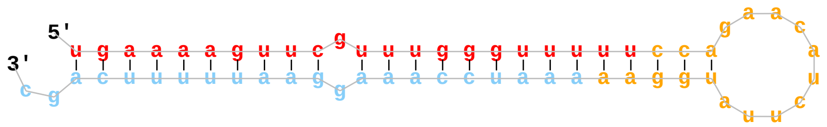

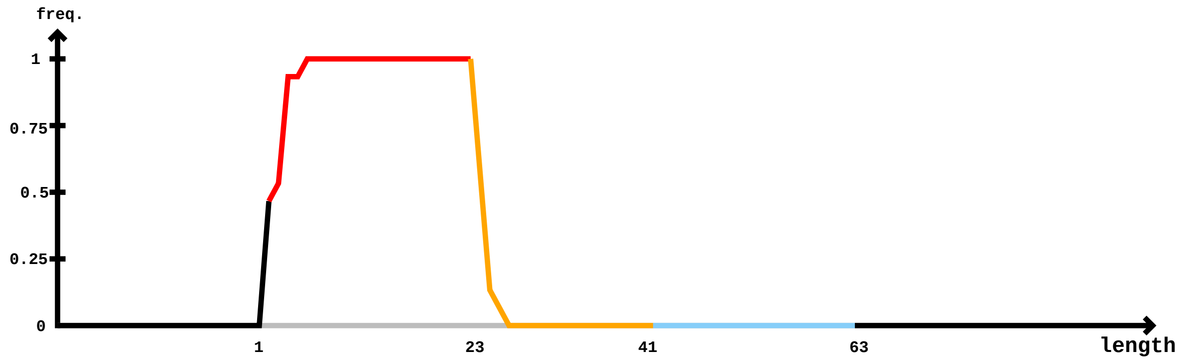

Mature   Star

```
5'- ucaaauaauauuggguuggcugaaaaguucguuuggguuuuuccagaacaucuuauggaaaaauccaaaggaauuuuucagccaacacaauauuaacuuucacagcuuugua   -3'    exp
    .....((((((((.((((((((((((((((.((((((((((((((..........)))))))))))))).)))))))))))))))).)))))))).................    reads    mm    sample
    ....................ugaaaaguucguuuggguuuuu......................................................................    7        0     seq
    .....................gaaaaguucguuuggguuuuu......................................................................    1        0     seq
    ......................aaaaguucguuuggguuuuuA.....................................................................    5        1     seq
    ......................aaaaguucguuuggguuuuucAa...................................................................    1        1     seq
    ........................aaguucAuuuggguuuuucc....................................................................    1        1     seq
```
